# The novel tankyrase inhibitor (AZ1366) enhances irinotecan activity in tumors that exhibit elevated tankyrase and irinotecan resistance

**DOI:** 10.18632/oncotarget.8626

**Published:** 2016-04-07

**Authors:** Kevin S. Quackenbush, Stacey Bagby, Wai Meng Tai, Wells A. Messersmith, Anna Schreiber, Justin Greene, Jihye Kim, Guoliang Wang, Alicia Purkey, Todd M. Pitts, Anna Nguyen, Dexiang Gao, Patrick Blatchford, Anna Capasso, Alwin G. Schuller, S. Gail Eckhardt, John J. Arcaroli

**Affiliations:** ^1^ Division of Medical Oncology, University of Colorado Denver and University of Colorado Cancer Center, Denver, CO, USA; ^2^ Division of Medical Oncology, National Cancer Centre Singapore, Singapore; ^3^ AstraZeneca R & D Boston, Massachusetts, Waltham, Massachusetts, MA, USA

**Keywords:** CRC, WNT, IRN, NuMA, PDTX

## Abstract

**Background:**

Dysregulation of the canonical Wnt signaling pathway has been implicated in colorectal cancer (CRC) development as well as incipient stages of malignant transformation. In this study, we investigated the antitumor effects of AZ1366 (a novel tankyrase inhibitor) as a single agent and in combination with irinotecan in our patient derived CRC explant xenograft models.

**Results:**

Six out of 18 CRC explants displayed a significant growth reduction to AZ1366. There was one CRC explant (CRC040) that reached the threshold of sensitivity (TGII ≤ 20%) in this study. In addition, the combination of AZ1366 + irinotecan demonstrated efficacy in 4 out of 18 CRC explants. Treatment effects on the WNT pathway revealed that tankyrase inhibition was ineffective at reducing WNT dependent signaling. However, the anti-tumor effects observed in this study were likely a result of alternative tankyrase effects whereby tankyrase inhibition reduced NuMA levels.

**Materials and Methods:**

Eighteen CRC explants were treated with AZ1366 single agent or in combination for 28 days and treatment responses were assessed. Pharmacokinetic (AZ1366 drug concentrations) and pharmacodynamic effects (Axin2 levels) were investigated over 48 hours. Immunohistochemistry of nuclear β-catenin levels as well as western blot was employed to examine the treatment effects on the WNT pathway as well as NuMA.

**Conclusions:**

Combination AZ1366 and irinotecan achieved greater anti-tumor effects compared to monotherapy. Activity was limited to CRC explants that displayed irinotecan resistance and increased protein levels of tankyrase and NuMA.

## INTRODUCTION

The Wnt signaling pathway plays a vital role in numerous biological processes, including embryonic development and the maintenance of adult stem cell populations [1]. In the colon, Wnt activation is essential in maintaining tissue homeostasis as it regulates the self-renewal of the stem cell population and commitment of progenitor cells to absorptive or secretory cell lineages [2]. The ultimate downstream effector of the Wnt pathway is β-catenin, a cytoplasmic protein whose stability is under the control of the APC destruction complex [1] consisting of glycogen synthase kinase 3β (GSK3β), casein kinase I (CKI), adenomatous polyposis coli (APC) and AXIN [3]. In the absence of canonical Wnt signaling, β-catenin levels are kept at low levels as it is continually phosphorylated by the destruction complex resulting in β-TrCP dependent polyubiquitination and subsequent proteasomal degradation. When the Wnt cascade is activated, the destruction complex is disassembled and β-catenin accumulates in the cytoplasm, ultimately leading to its nuclear translocation and TCF/LEF dependent transcription of Wnt target genes [1].

CRC is the second most deadly cancer in the United States with an estimated 49,700 deaths in 2015 [4]. Although highly curable if detected early, patients with advanced disease have an overall 5-year survival rate of 11%, making this an unmet need in drug development. The role of the Wnt pathway in the development of colorectal cancer was first discovered over two decades ago [5], and it is well established that WNT pathway dysregulation is one of the earliest events in the initiation of colorectal cancers [6]. The majority of CRCs harbor a mutation in either the APC or CTNNB1 gene [7], and these mutations lead to accumulation of β-catenin and subsequent up-regulation of Wnt target genes. This aberrant genetic event facilitates the proliferation of colonic crypt progenitors, which eventually results in oncogenic transformation after these progenitors acquire additional genetic insults [1]. Interestingly, a recent study has demonstrated that restoration of normal APC function in CRC cells cause the cells to differentiate and regress, thus validating the Wnt pathway as an attractive target for the treatment of CRC [8].

The scaffolding protein Axin has been shown to be the rate-limiting component of the APC destruction complex and found to regulate the efficiency of the β-catenin destruction complex [9]. Axin is regulated by tankyrase, a PARP family enzyme that PARyslates Axin targeting it for proteasomal degradation [10]. It was initially shown that inhibition of tankyrase stabilized Axin and subsequently downregulated β-catenin mediated transcription [11, 12]. Since that time, the development of tankyrase inhibitors has been an intense area of interest in several types of cancer, particularly CRC [13–16]. In addition to regulating the WNT pathway, tankyrase has been involved in controlling the function of many other biological processes potentially enhancing the clinical utility of tankyrase inhibitors. Considering that the studies investigating tankyrase inhibitors in CRC have been conducted on a very limited number of CRC cell lines and explants, we set out to determine the efficacy of a novel tankyrase inhibitor (AZ1366) [17] as a single agent and in combination with irinotecan in 18 unique CRC PDTX models.

## RESULTS

### AZ1366 treatment effects in a CRC PDTX model

**Figure 1:**
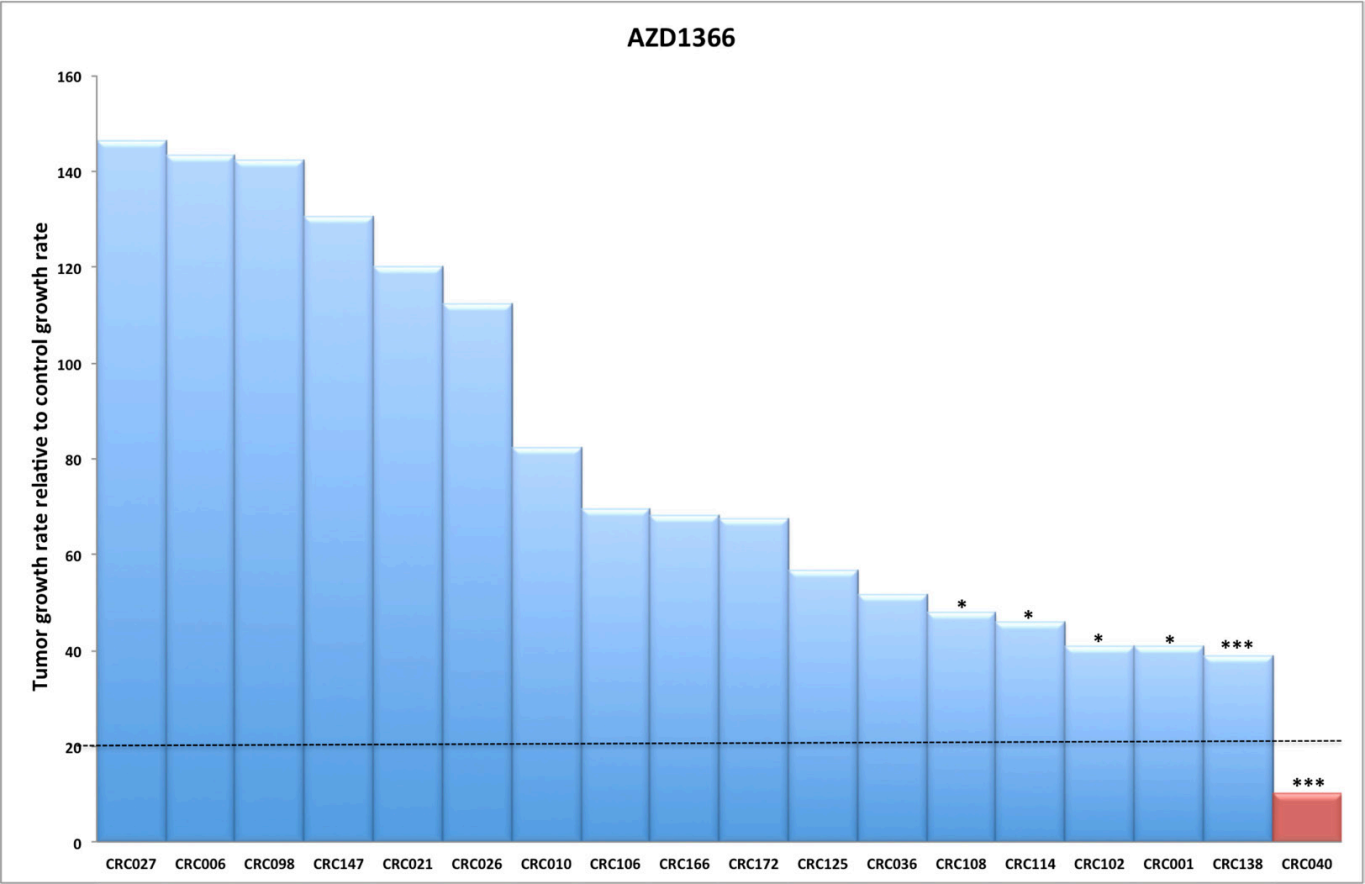
The efficacy of AZ1366 on tumor growth in CRC explants (**A**) Eighteen CRC explants were treated with AZ1366 50 mg/kg daily × 5 days/week for 28 days. A significant reduction in tumor growth was observed in six CRC explant models (CRC108, 114, 102, 001, 138 and 040). Columns, mean (*n* = 10 tumors per group). Significance *p < 0.05; ***p < 0.001.

To determine the efficacy of a novel WNT tankyrase inhibitor (AZ1366), we explored treatment effects on tumor growth on 18 unique CRC PDTX models. Table 1 shows the patient characteristics and mutational status of RAS, RAF, PIK3CA, APC and CTNNB1 of these 18 tumors. There were 11 CRC explants with mutations in the APC gene and 7 CRC explants that were APC wild type. We observed a significant treatment effect on 6 out of 18 (33%) CRC explants (CRC108, 114, 102, 001, 138, 040) at the end of study (Figure 1). A TGII of < 20% (> 80% tumor growth inhibition) was chosen as a cut-off for progressive disease. Using this stringent measure, only CRC040 (TGII = 10%) was considered sensitive while CRC 138, 001, 102, 114 and 108 (TGII > 20%) were determined to have intermediate sensitivity to AZ1366.

**Table 1:** Patient characteristics and molecular features of PDTX models

Specimen ID	Colon or Rectal	Age at consent	Primary or metastatic	Previous Chemotherapy/Treatment	Stage	KRAS	NRAS	BRAF	PIK3CA	APC	CTNNB1
**CRC-001**	Colon	69	Primary	Oxaliplatin and bevacizumab	IV	Mut	WT	WT	WT	Mut	WT
**CRC-006**	Colon	42	Primary	Capecitabine+ radiation, FOLFOX, bevacizumab, and irinotecan + cetuximab	IV	Mut	WT	WT	WT	Mut	WT
**CRC-010**	Rectal	52	Primary	No	III	WT	WT	WT	WT	WT	WT
**CRC-021**	Rectal	71	Primary	No	II	Mut	WT	WT	WT	WT	WT
**CRC-026**	Colon	48	Metastatic	FOLFOX and bevacizumab	IV	WT	Mut	WT	WT	Mut	WT
**CRC-027**	Colon	56	Metastatic	No	IV	Mut	WT	WT	WT	Mut	WT
**CRC-036**	Rectal	42	Metastatic	Xeloda + radiation, FOLFOX, irinotecan + cetuximab	IV	Mut	WT	WT	WT	Mut	WT
**CRC-040**	Rectal	63	Primary	No	II	Mut	WT	WT	Mut	Mut	WT
**CRC-098**	Colon	50	Primary and Metastatic	No	IV	Mut	WT	WT	Mut	Mut	WT
**CRC-102**	Rectal-Sigmoid	55	Metastatic	FOLFOX	IV	Mut	WT	WT	WT	WT	WT
**CRC-106**	Sigmoid	57	Metastatic	FOLFOX + Avastin	IV	WT	WT	WT	WT	WT	WT
**CRC-108**	Sigmoid	44	Metastatic	CAPE, Ox, Avastin	IV	Mut	WT	WT	WT	Mut	WT
**CRC-114**	Colon	70	Primary	No	III	WT	WT	Mut	WT	Mut	WT
**CRC-125**	Rectal	58	Metastatic	Yes- but don't know chemo regimen	IV	WT	WT	WT	WT	WT	WT
**CRC-138**	Colon	78	Metastatic	No	IV	Mut	WT	WT	WT	WT	WT
**CRC-147**	Rectal-Sigmoid	77	Primary and Metastatic	No	IV	WT	WT	WT	WT	Mut	WT
**CRC-166**	Rectal	41	Metastatic	FOLFOX + AVASTIN	IV	WT	WT	WT	WT	Mut	WT
**CRC-172**	Sigmoid	50	Metastatic	FOLFOX	III	WT	WT	WT	WT	WT	WT

### Pharmacokinetic and pharmacodynamic relationship of AZ1366 in the sensitive CRC040 explant

**Figure 2:**
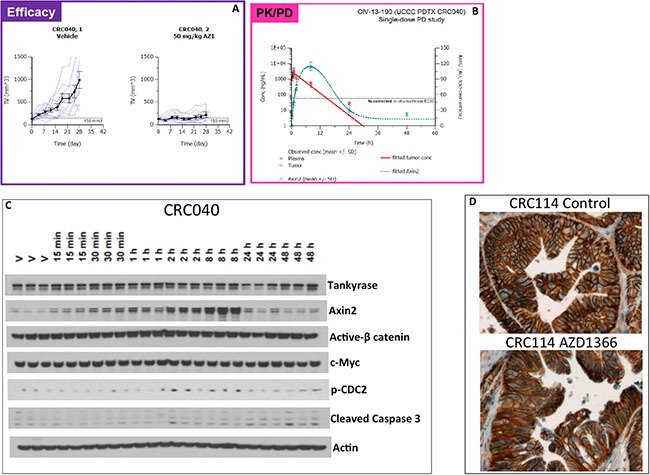
Pharmacokinetic and pharmcodynamic analysis of AZ1366 on the sensitive CRC040 (**A**) Tumor growth kinetics of individual tumors between vehicle and AZ1366 treated mice. (**B**) Pharmacokinetic (plasma and tumor) and pharmacodymanic (Axin2) relationship after a single dose of AZ1366. Plasma and tumor were obtained at 0 hr, 0.25 hr, 0.5 hr, 1 hr, 2 hr, 8 hr, 24 hr and 48 hrs after AZ1366 administration. A peak in plasma and tumor concentration of drug occurred before 1 hour, while Axin2 stabilization occurred at 8 hr following treatment. (**C**) Western blot analysis of proteins following AZ1366 treatment in mice. No changes were seen in tankyrase, active β-catenin and c-myc throughout the time course examined. In contrast, an increase in Axin2, p-CDC2 and cleaved caspase 3 were elevated as a result of tankyrase inhibition. (**D**) Representative depiction of β-catenin immunohistochemistry in CRC114 at the end of study.

Drug concentrations of AZ1366 were quantified in the plasma and tumor over a 48-hour time period in mice bearing CRC040, where AZ1366 exhibited potent anti-tumor growth kinetics in all AZ1366 tumor-bearing mice when compared to vehicle (Figure 2A). As illustrated in Figure 2B, both plasma and tumor samples showed similar mean concentrations of AZ1366, with a peak occurring before an hour following one oral dose of AZ1366 at a concentration of 50 mg/kg. Rapid decreases in AZ1366 drug concentrations were seen in plasma and tumor after one hour following AZ1366 administration with undetectable levels occurring after 30 hours of treatment (Figure 2B). Next, we assessed AZ1366 effects on Axin2 stabilization over the same time frame. We demonstrated a significant increase in Axin2 stabilization as early as 15 minutes with peak levels occurring 8 hours after dosing (Figure 2B–2C). Mean concentrations of Axin2 protein levels were reduced after 8 hours of dosing. Given that Axin2 is a member of the β-catenin destruction complex and stabilized after tankyrase inhibition, we set out to determine whether treatment led to a reduction in β-catenin. While Axin2 was significantly elevated after treatment, no marked decrease in active β-catenin was observed. Similar results were seen in c-myc (a WNT target gene) where no changes occurred during the time course that we investigated. In contrast, an increase in the phosphorylation of CDC2 and cleaved caspase 3 were seen at 8 hours and 48 hours respectively. Finally, immunostaining of nuclear β-catenin was evaluated on the CRC 114 explant. Although treatment with AZ1366 significantly reduced tumor growth in this explant, there was no decrease in nuclear β-catenin after treatment (Figure 2D). These results demonstrate that AZ1366 is a potent stabilizer of Axin2; however, a lack of β-catenin degradation suggests that alternative tankyrase inhibition mediated effects may be responsible for facilitating the anti-tumor properties of this compound.

### Investigation of the efficacy of AZ1366 + irintoecan on tumor growth in a CRC explant model

**Figure 3:**
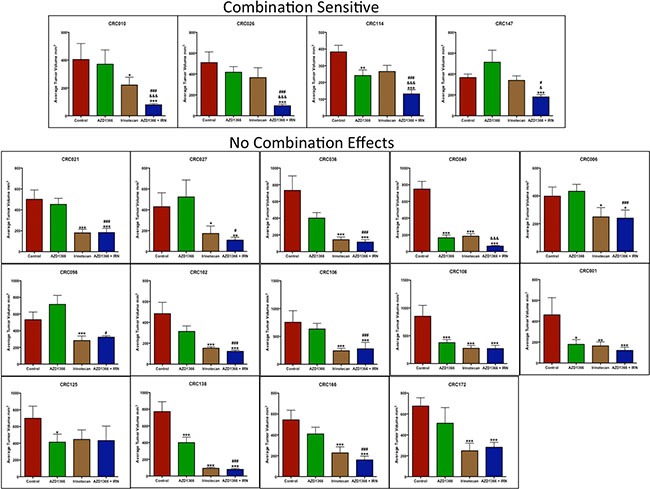
Anti-tumor activity of AZ1366 alone and in combination with irinotecan in 18 CRC patient-derived tumor xenograft models Plotted is tumor volume at the end of study. Four CRC explants (CRC010, 026, 114 and 147) displayed a significant combination treatment response when compared to single agent AZ1366 or irinotecan. *p < 0.05, ***p < 0.001 when compared to control, ^#^*p* < 0.05, ^###^*p* < 0.001 when compared to AZ1366, ^&^*p* < 0.05, ^&&&^*p* < 0.001 when compared to irinotecan.

In this study, we also assessed the addition of irinotecan (a standard of care agent used in CRC) in combination with AZ1366 in 18 CRC explant models. As displayed in Figure 3, we observed a significant combination treatment effect (analysed at the end of study) in 4 CRC explants that included CRC010, CRC026, CRC114 and CRC147. The greatest combination effects were seen in CRC010, CRC026 and CRC147 which all displayed resistance to single agent AZ1366. All other CRC explants demonstrated either no treatment effects in all groups or a combination treatment effect that was not significantly different from single agent AZ1366 and/or irinotecan (Figure 3).

### AZ1366 stabilizes Axin2 but does not facilitate degradation of β-catenin and a decrease in WNT dependent transcription

**Figure 4:**
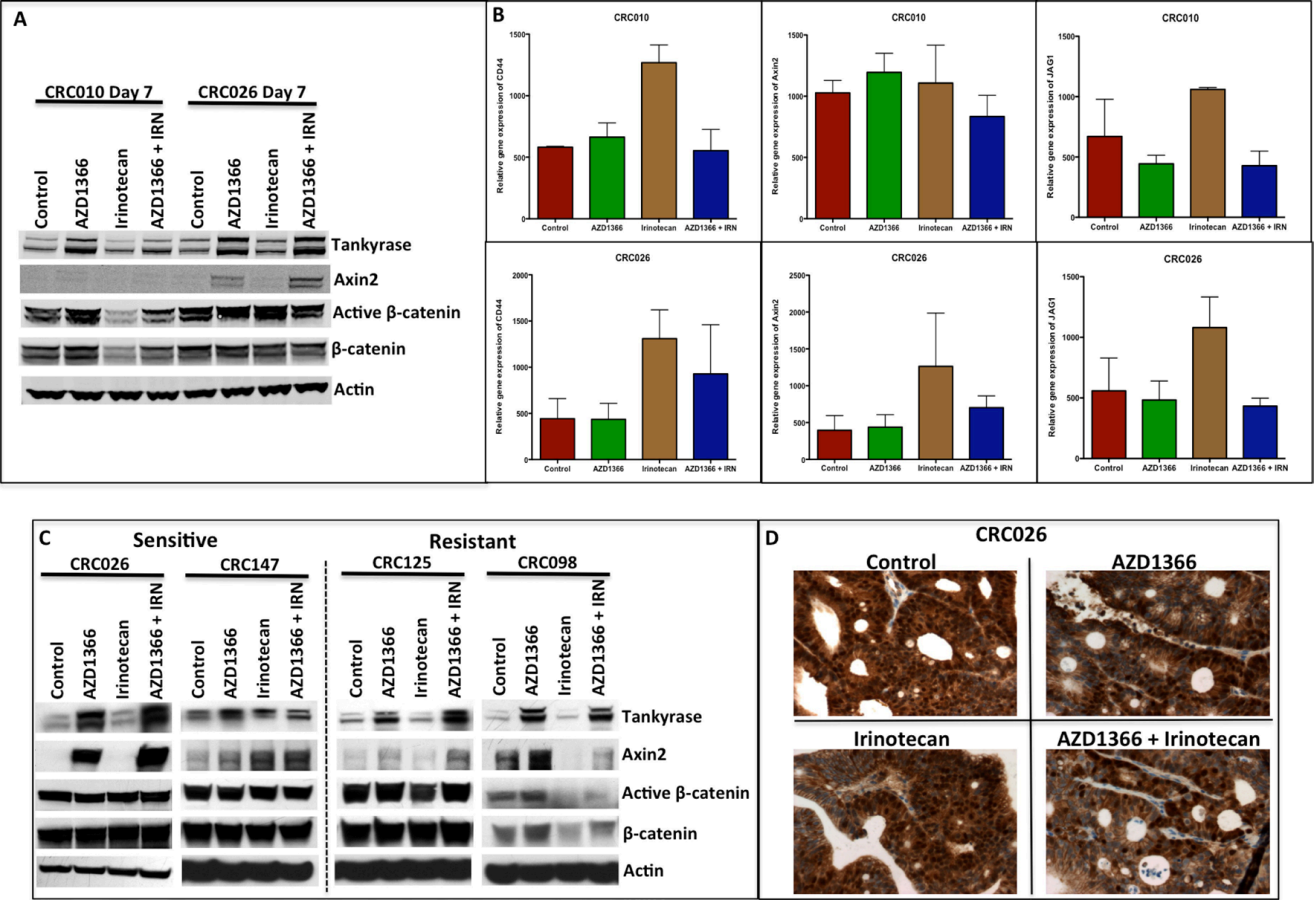
Effects of single agent and combination on the WNT/β-catenin singling pathway (**A**) Western blot analysis of expression of members of Wnt/β-catenin (Tankyrase, Axin2, active β-catenin and β-catenin) after 7 days of single agent and combination treatment. While AZ1366 and AZ1366 + IRN groups exhibited Axin2 stabilization, no decrease was seen in active β-catenin and β-catenin. (**B**) RT-PCR analysis of the WNT dependent genes CD44, Axin2 and JAG1 at day 7 of treatment in CRC010 and CRC026 showed no significant changes in the AZ1366 or AZ1366 + irinotecan groups. (**C**) Evaluation of the Wnt/β-catenin pathway (Tankyrase, Axin2, active β-catenin and β-catenin) at the end of study in the combination sensitivity CRC026/CRC147 and no combination effects CRC125/098. Axin2 was stabilized in the AZ1366 and AZ1366 + IRN groups, but treatment failed to reduce levels of active β-catenin and β-catenin. (**D**) Representative illustration of β-catenin immunohistochemistry in CRC026 at the end of study after AZ1366, irinotecan and AZ1366 + irinotecan.

In an effort to understand the antitumor properties of AZ1366 + irinotecan, we evaluated treatment effects on the WNT signaling pathway on the combination-sensitive CRC explants 010 (APC wt/CTNNB1 wt) and 026 (APC mut/CTNNB1 wt). Treatment with AZ1366 and AZ1366 + irinotecan resulted in an increase in Axin2 protein levels after 7 days of treatment (Figure 4A), confirming that the inhibition of tankyrase by AZ1366 leads to Axin2 stabilization. Interestingly, we observed an elevation of tankyrase in the AZ1366 treatment groups suggesting a compensatory mechanism whereby tankyrase gene expression is increased as a result of tankyrase inhibition. Similar to the findings of single agent AZ1366 on CRC040 and CRC114, no differences were observed in CRC010 and CRC026 with treatment in protein levels of active β-catenin (Figure 4A) as well as in WNT dependent gene transcription of CD44, Axin2 and Jag1 (Figure 4B). Comparable results were found at the end of study in the sensitive CRC026 and CRC147 and the resistant CRC125 explants; an increase in Axin2 was identified following AZ1366 treatment (single agent and combination), but no changes in active β-catenin were observed in all treatment groups (Figure 4C). Immunostaining of nuclear β-catenin confirmed that AZ1366 does not enhance the degradation of β-catenin as there were no significant differences in nuclear staining after AZ1366 and AZ1366 + irinotecan treatment (Figure 4D).

### Tankyrase inhibition decreases NUMA and facilitates G_2_M arrest

**Figure 5:**
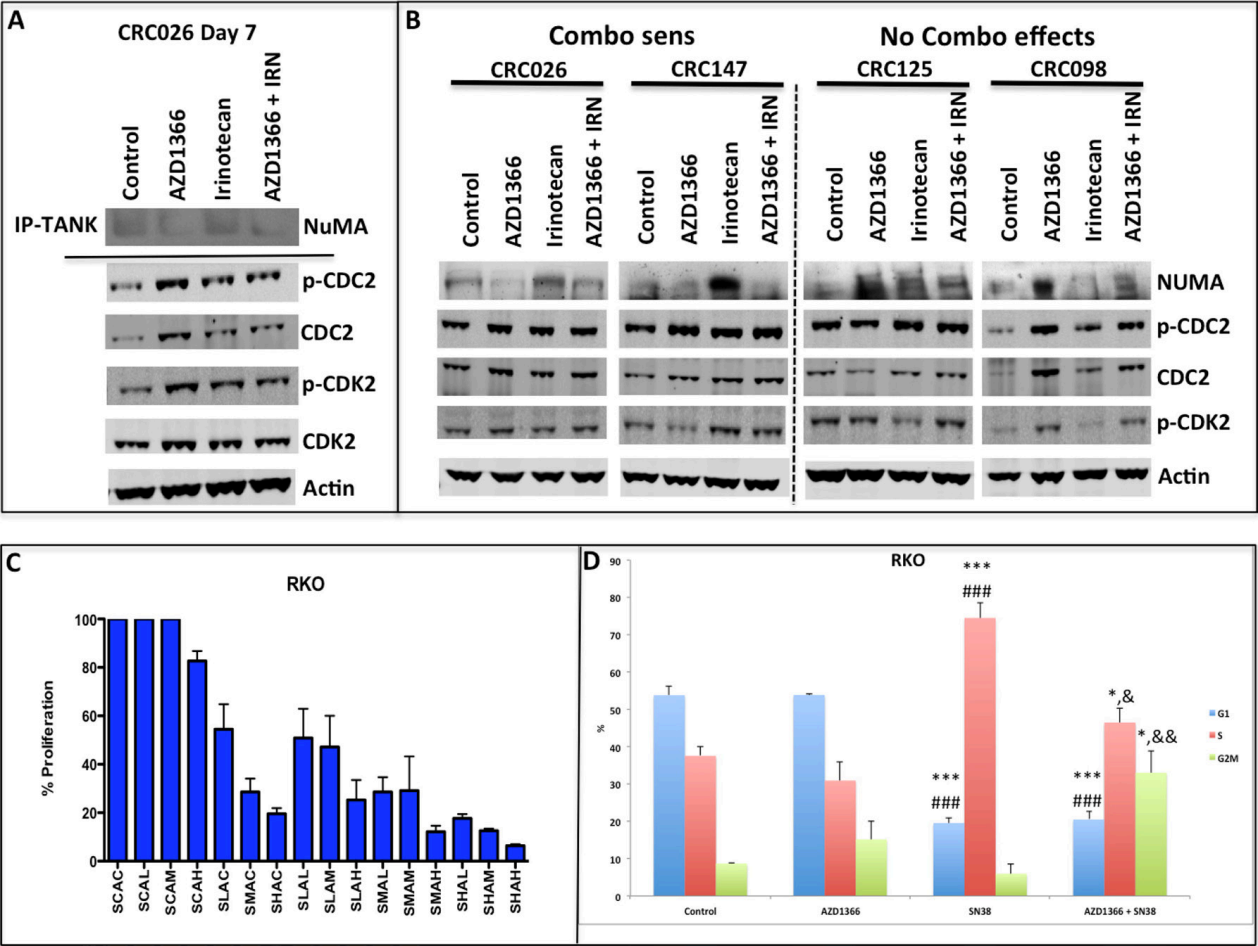
Analysis of treatment effects of single agent and combination on NuMA and G_2_M proteins (**A**) Immunoprecipitation of tankyrase and probing of NuMA and western blot determination of CDC2 and CDK2 on the CRC 026 combination sensitive explant 7 days after treatment. AZ1366 and AZ1366+ irinotecan reduced the interaction between NuMA and tankyrase. In addition, an increase in the activation CDC2 and CDK2 was observed in all treatment groups. (**B**) Evaluation of NuMA, CDC2 and CDK2 at the end of study. A decrease in NuMA was seen in the AZ1366 and combo groups in the combo sensitive CRC explants, while an increase in NuMA was seen in the CRC explants where no combination effects were observed. (**C**) The effects of combinational therapy on the RKO CRC cell line *in vitro*. The RKO exhibited combination sensitivity to AZ1366 and SN-38. (**D**) Cell cycle analysis of RKO 24 hours after treatment. The RKO cell line displayed an S phase and G_2_M phase % increase after combinational treatment. *p < 0.05, ***p < 0.001 when compared to control, ^###^*p* < 0.001 when compared to AZ1366, ^&^*p* < 0.05, ^&&^*p* < 0.01 when compared to irinotecan.

Given that AZ1366 inhibits tankyrase activity but does not modulate downstream WNT signalling in this study, we set out to examine alternative mechanisms of action to explain the tumor growth inhibition of AZ1366. In addition to regulating the WNT signaling pathway, tankyrase has been described to function in regulating telomere length by interacting with TRF1 as well as separation of sister chromatids during mitosis by binding to NuMA, a nuclear mitotic apparatus protein [18–20]. Therefore, we examined the treatment effects of tankyrase inhibition on levels of TRF1 and NuMA. Whereas no treatment effect on TRF1 was seen at days 7 and 28 in all the treatment groups when compared to control (data not shown), a decrease in NuMA was observed in the sensitive CRC026 after treatment with AZ1366 and AZ1366 + irinotecan (Figure 5A and 5B). In particular, tankyrase and NuMA interaction as demonstrated by co-immunoprecipitation revealed a decrease in binding of tankyrase to NuMA in CRC026 after AZ1366 treatment (single agent and combination) at day 7. Additionally, AZ1366 and AZ1366 + irinotecan reduced NuMA levels at the end of study in the sensitive CRC026 and CRC147 explants. Both experiments indicate that tankyrase may be important in regulating NuMA during mitosis. To further delineate the effects of treatment on cell cycle, we performed western blot on CDK2 (S and G_2_M) and CDC2 (G_2_M), whereby phosphorylation of these protein inhibit cell cycle progression. As shown in Figure 5A and 5B, a significant increase the phosphorylation of CDC2 and CDK2 was identified in CRC026 after 7 days of treatment but not at the end of study. An increase in p-CDC2 and p-CDK2 was also seen in the sensitive CRC147 following AZ1366 + irinotecan at the end of study. In order to characterize the potent combination effects on cell cycle seen *in vivo*, we treated with AZ1366, SN38 (active metabolite to irinotecan) and AZ1366 + SN38 on 8 CRC cells (data not shown). Only the RKO (APC wt) (Figure 5C) and LoVo (APC mut) (data not shown) cells showed a combination treatment effect. Cell cycle analysis of RKO showed an increase in % cells in S and G_2_M phase after treatment with AZ1366 + SN38 (Figure 5D). Together, these data indicate that the potent combination effects observed in this study are likely due to inhibiting both S phase progression by irinotecan and G_2_M progession by AZ1366 further preventing the ability of tumor cells from overcoming this inhibition.

### Combination sensitive tumors exhibit elevations in tankyrase and NuMA expression and demonstrate irinotecan resistance

**Figure 6:**
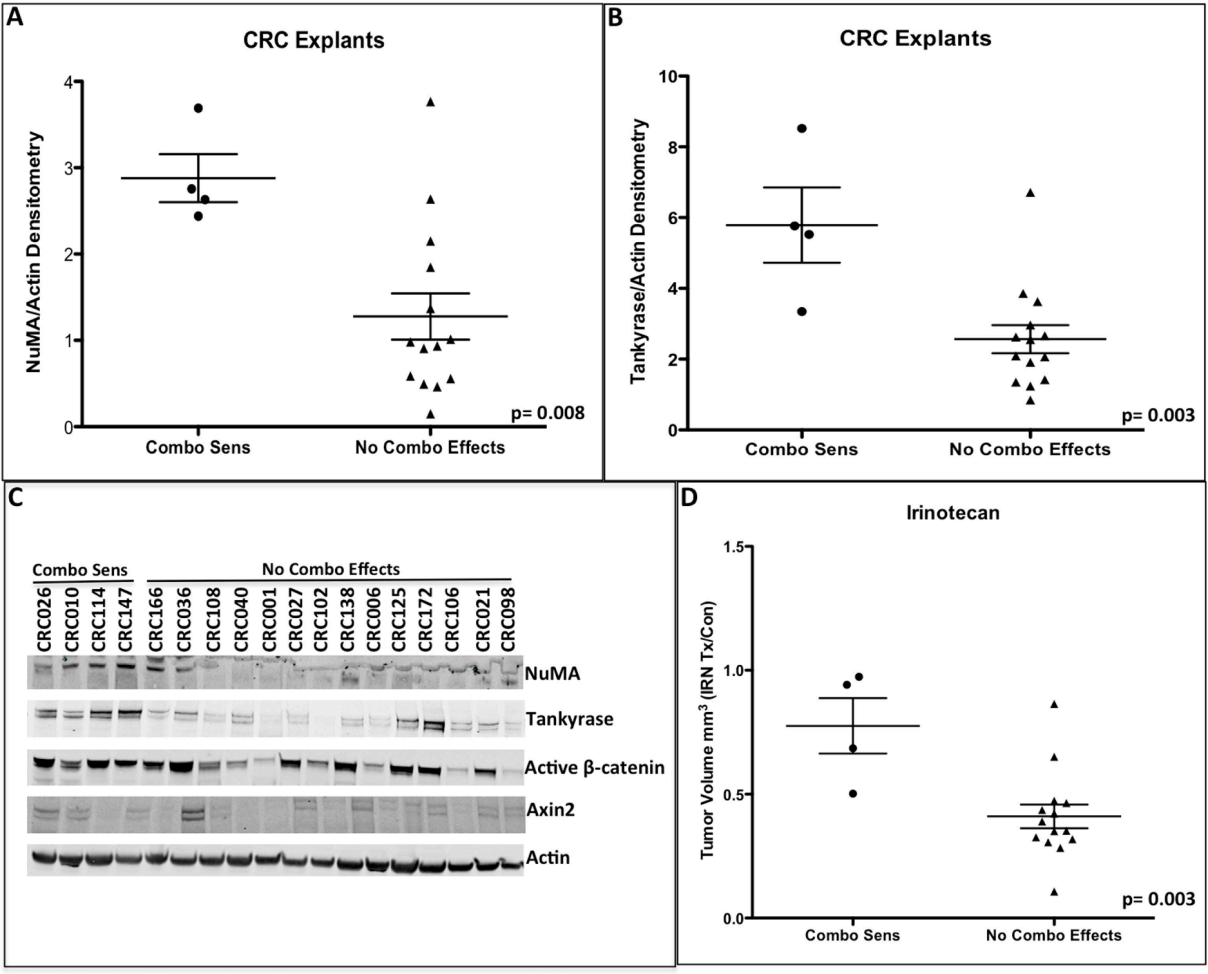
Baseline NuMA (A) and tankyrase (B) protein levels were significantly increased in the combinational sensitive CRC explants (**C**) A representative immunoblot of NuMA, tankyrase, active β-catenin and Axin2. (**D**) Comparison of irinotecan response between combinational sensitive CRC explants vs. CRC explants that did not exhibit any combinational treatment effects. Combinational sensitive CRC explants displayed resistance to irinotecan therapy.

Finally, we sought to explore if baseline tumor tankyrase and NuMA levels were associated with sensitivity to combination treatment. As illustrated in Figure 6A–6B, a significant increase in tankyrase and NuMA protein levels were seen in tumors that displayed combination sensitivity (4 combo sensitive tumors) when compared to tumors where there were no combination effects (14 tumors). Figure 6C shows a representative blot of tankyrase and NuMA on all 18 CRC explants that were examined. Interestingly, there were no differences observed between these groups (combo sensitive vs no combo effects) with respect to Axin2 and active β-catenin (Figure 6C). Moreover, we identified an association with CRC explants that exhibited a significant combination treatment effect and resistance to single agent irinotecan (Figure 6D).

## DISCUSSION

Colorectal cancer is initiated by dysregulation of the canonical WNT signaling cascade whereby mutations in the APC or CTNNB1 gene potentiate tumorigenesis in the colon [7, 21–23]. β-catenin is the focal point of this pathway that is responsible for specific activation of WNT dependent transcription of genes that are involved in enhancing cellular proliferation, survival and metastasis [24]. Even in the presence of other mutations that accumulate during oncogenic transformation, WNT signaling has been determined in some CRC models to be important in driving tumor growth (8). Given the importance of this pathway in the development and proliferation of CRC, there is a concerted effort in targeting the WNT pathway to improve patient survival. Recently developed tankyrase inhibitors have shown promise at inhibiting WNT dependent signaling by stabilizing Axin, a essential component of the destruction complex, which leads to the proteosomal degradation of β-catenin in the nucleus [11, 12, 16]. These observations led us to explore a novel WNT tankyrase inhibitor in a panel of CRC PDTX models.

In evaluating AZ1366 single agent efficacy in our CRC PDTX models, we identified a significant reduction in tumor growth in 6 (2 APC wild type and 4 APC mutant) out of 18 unique models. Despite these findings, using a stringent TGII cut-off of < 20% (> 80% tumor growth inhibition) as previously described by our group [25, 26], only CRC040 reached this threshold indicating sensitivity to AZ1366. This cut-off we believe represents a more clinically relevant level of sensitivity similar to RECIST criteria used in the clinic. Several other tankyrase inhibitors (G007-LK, XAV939 and NVP-TNKS656) have been evaluated in preclinical models of CRC and have demonstrated limited activity [12, 16, 27]. For instance, the tankyrase inhibitor G007-LK was shown to have anti-tumor effects in 2 out of 4 CRC cell lines [16]. Specifically, G007-LK attenuated tumor growth in the COLO320 and SW403 cell lines in a xenograft model. In contrast, no treatment effects were observed with G007-LK in the HCT-15 and DLD-1 cell lines. In a separate study, XAV939 significantly reduced the number of colonies in the DLD-1 cell line in a colony formation assay [12]. More recently, the tankyrase inhibitor NVP-TNKS656 was shown to significantly inhibit tumor growth in 1 out of 3 CRC PDTX models [27]. Together, these results suggest that tankyrase inhibition has limited single agent activity in CRC. There are several possible explanations for these findings. First, although the WNT pathway is implicated in the development of CRC, WNT signaling may not be a driver of tumor growth in the majority of CRC tumors at later stages of progression. A second possibility is that targeting the WNT pathway upstream may not be as efficient at reducing β-catenin levels since APC/CTNNB1 mutations occur further downstream. Further genetic knockout studies in PDTX models as well as compounds that specifically target WNT/β-catenin dependent transcription will provide better insight into tumor dependence on the WNT/β-catenin pathway.

Since AZ1366 demonstrated efficacy in a subset of CRC tumors, we set out to determine the pharmacokinetic and pharmacodynamic parameters of AZ1366 on the sensitive CRC040 explant. Although we observed high concentrations of drug levels in tumor 1 hour after AZ1366 administration and robust Axin2 stabilization at 8 hours, we were not able to demonstrate any decrease in active β-catenin or c-Myc, a WNT dependent gene, at all time points examined. Similar results were seen in the APC mutant sensitive CRC114 explant where treatment with AZ1366 did not decrease nuclear staining of β-catenin. A study by Lau et al. [16] assessed the ability of the tankyrase inhibitor G007-LK to decrease WNT/β-catenin signaling in 11 APC mutant CRC cell lines *in vitro*. They showed a reduction in WNT/β-catenin in 6 of the CRC cell lines that ranged from 29%–76% decrease in WNT activated genes. In contrast, treatment with G007-LK failed to inhibit WNT/β-catenin signaling in 5 of the CRC cell lines [16]. They also determined that the location of the APC mutation did not correlate with sensitivity to this compound [16]. Considering these findings, tankyrase inhibition may not always be effective at reducing WNT/β-catenin signaling in tumors that harbor mutations in the APC gene and that alternative tankyrase dependent cellular effects may be responsible for the anti-tumor properties of tankrase inhibition in sensitive tumors where WNT/β-catenin signaling is unchanged. Indeed, we demonstrate an elevation in the activation of the G_2_M protein CDC-2 resulting in an induction of apoptosis evident by an increase in cleaved caspase 3 that supports this notion. Unravelling the mechanisms underlying tumor response to tankyrase inhibition will yield a better understanding of whether WNT signaling or other tankyrase interactions are responsible for tumor growth inhibition in CRC.

Irinotecan is used as standard therapy for the treatment of CRC [28, 29]. Although treatment responses are observed in patients, drug resistance remains a major problem ultimately leading to disease recurrence and death [28, 29]. It has been shown that Wnt pathway inhibition may facilitate sensitivity to irinotecan and overcome treatment resistance [30]. In our study, as expected, we observed a range of irinotecan responses in our CRC PDTX models from sensitive to resistant. The addition of the tankyrase inhibitor AZ1366 to irinotecan significantly reduced the growth of tumors in 4 of our CRC PDTX models. Interestingly, tumors that were responsive to combination therapy were significantly more resistant to single agent irinotecan therapy when compared to CRC explants where a combination effect was not observed. A study by Gurney and colleagues [31] also showed combination effects of OMP-18R5 (an anti-Frizzled receptor antibody) + irinotecan in a CRC PDTX model. The effects that were detected were in an APC/β-catenin wild type tumor. We demonstrated a combination effect in both APC/β-catenin wild type (CRC010) and APC mutant (CRC026, CRC114 and 147) tumors. These results imply that tankyrase may be important at mediating resistance to irinotecan therapy and inhibition of tankyrase may augment tumor death in a subset of CRC tumors.

As reported in our single agent AZ1366 studies, we considered that the combination effects we demonstrated may not be a result of the WNT signaling pathway mediating resistance to irinotecan. Therefore, we first examined treatment effects on the WNT/β-catenin signaling pathway. Similar to the results identified in the single agent experiments, AZ1366 and AZ1366 + irinotecan enhanced Axin2 stabilization; however, there was no reduction in β-catenin (active or nuclear β-catenin) or WNT dependent signaling after treatment in the combination sensitive CRC explants. These findings led us to explore the interaction between tankyrase and other proteins that play an important role in cellular proliferation and survival. In particular, we focused our efforts in assessing the effects of AZ1366 on NuMA. NuMA is a key component of the mitotic spindle assembly apparatus that plays a functional role in organizing and stabilizing microtubules during mitosis [32, 33]. Tankyrase is responsible for PARsylating NuMA and has been shown to be important in normal spindle formation, as tankyrase knockout experiments have revealed defects in the assembly of the mitotic spindles [34]. The results of this investigation revealed that tankyrase inhibition (AZ1366 and AZ1366 + irinotecan) resulted in a decrease in the interaction between tankyrase and NuMA as well as protein levels of NuMA. These findings indicate that tankyrase blockade may prevent the segregation of sister chromatids during mitosis by reducing NuMA function or levels during this critical step in cell division. This was evident since we observed an increase in the activation of the G_2_M protein CDC2 preventing G_2_M progression. Although further work is necessary to determine if this alternative mechanism is responsible for the treatment effects, these findings support the idea that inhibition of S phase by irinotecan and G_2_M phase by tankyrase inhibition are likely responsible for generating potent anti-tumor effects in combination groups observed in this study.

Since combination efficacy was limited to a subset of tumors treated, we set out to determine whether we could identify a predictive biomarker of sensitivity that may explain the enhanced combination sensitivity in these tumors. Interestingly, we found that baseline tankyrase and NuMA protein levels were significantly elevated in tumors that responded to combination therapy. Additionally, an association between combination sensitivity and irinotecan resistance was evident. These findings indicate that tankyrase regulation of NuMA may be responsible for conferring resistance to irinotecan therapy. While in this study it is difficult to ascertain the relationship between tankyrase and NuMA levels with irinotecan resistance, we postulate that NuMA may be an important mediator in irinotecan resistance by promoting the progression of cells through mitosis.

In summary, this study revealed that combination therapy proved to be effective in a subset of CRC tumors that exhibit resistance to irinotecan therapy as well as elevated levels of tankyrase and NuMA. Although the mechanism involved in the anti-tumor properties of the tankyrase inhibitor (AZ1366) in this study appears not to implicate the WNT/β-catenin signaling pathway, it is intriguing that alternative tankyrase mechanisms may be responsible for overcoming irinotecan therapy resistance in a subset of CRC tumors. These findings support further investigation of this combination therapy for the treatment of CRC patients.

## MATERIALS AND METHODS

### CRC patient derived tumor xenograft model

Patient-derived colorectal adenocarcinoma tumor specimens were obtained from consenting patients at the University of Colorado Hospital in accordance with protocols approved by the Colorado Multiple Institutional Review Board. Four-to-six week old female athymic (nu^+^/nu^+^) mice were obtained from Harlan laboratories (Washington DC) under an approved research protocol by the Institutional Animal Care and Use Committee. The tumor pieces were implanted in mice and expansion of the F1-F3 generations was carried out as previously described [35–37]. Tumors were expanded in the left and right flanks of 5-6 mice (10 evaluable tumors per group). Mice were randomized into vehicle, AZ1366, irinotecan or AZ1366 + irinotecan groups when tumor volumes reached ∼200 mm^3^. Mice were treated daily with AZ1366 (50 mg/kg-daily × 5 days) by oral gavage and/or irinotecan (15 mg/kg-weekly) by ip for 28 days. Mice were monitored daily for signs of toxicity and tumor size was evaluated twice per week by caliper measurements using the following formula: tumor volume = [length × width^2^] * 0.52.

### Pharmacokinetic and pharmacodynamic analysis of AZ1366

The CRC040 tumor was expanded in mice as described above. Animals were euthanized at 15 minutes, 30 minutes, 1 hour, 2 hours, 8 hours, 24 hours and 48 hours after a single dose of AZ1366 at a concentration of 50 mg/kg. Plasma was separated by centrifugation and frozen at −80°C. Tumors were removed from mice and divided into a tube for PK and PD analyses. Both tumors were frozen on dry ice immediately after removal from the mouse and then stored at −80°C.

Plasma samples were analysed for parent only using a protein precipitation extraction procedure, followed by LC-MS/MS analysis. Ten calibration standards were prepared in blank plasma with concentration range of 1.0 ng/mL to 10,000 ng/mL. Three quality control samples at 25, 250 and 2,500 ng/mL were also included in the analysis. 50 μL standard or unknown plasma samples in a 96-well sample plate were mixed with 250 μL acetonitrile containing an internal standard. After centrifugation, 225 μL of the supernatant was transferred to a new plate. Samples were dried under nitrogen and then reconstituted using 250 μL 80:20 mobile phase A: mobile phase B solvent. Aliquots of 20 μL reconstituted samples were injected into a Waters XBridge C18 3.5 um, 30 × 3 mm column. Mobile phases included 10 mM ammonium formate with 0.1% formic acid (mobile phase A) and acetonitrile with 0.1% formic acid (mobile phase B) with a gradient starting at 15% B and held for 0.5 minute, then linear increased to 95% B over next 1 minute. LC-MS/MS analysis was conducted using an API 4000 triple quadrupole mass spectrometer and processed with Analyst (AB Sciex Instruments). Selected reaction monitoring of m/z 417.2 to m/z 341.2 was utilized for the detection of AZ13401366 in the LC-MS/MS.

For PD analysis, tumors were lysed and immunoblotting of the proteins tankyrase, Axin2, active- β-catenin, c-Myc, p-CDC2, cleaved caspase 3 and Actin (Cell Signaling, Danvers, MA) were performed as described below.

### Immunoprecipitation

Equal amounts of protein and volume in each group were used for the immunoprecipitation procedures. Five microliters of anti-tankyrase (Santa Cruz Biotech, Santa Cruz, CA) was added to each sample followed by incubation (rotator) at room temperature for 1 hour. After 1 hour, 25 μl of protein G magnetic beads (Invitrogen-Dynal) was added to each sample and incubated for an additional 30 minutes. The samples were boiled for 5 minutes and loaded onto a 4–12% Bis-Tris precast gel. NuMA rabbit polyclonal antibody (Cell Signaling, Danvers, MA) was used for detection at a dilution of 1:1,000.

### Immunoblotting

Tumor tissues (15 mg/tumor tissue) were minced on ice and homogenized using a Qiagen tissue lyser and centrifuged at 16,000 g at 4°C for 10 min. The total protein in samples was determined using the 660 Protein Assay kit. Forty micrograms of sample were electrophoresed on 4–12% Bis-Tris precast gels (Life Technologies, Carlsbad, CA). After electrotransfer to an iBlot nitrocellulose membrane (Life Technologies, Carlsbad, CA), membranes were blocked at room temperature with TBS [10 mmol/L Tris-HCl (pH 7.5), 0.5 mol/L NaCl, and 0.1% (v/v) Tween 20] containing 5% nonfat milk (BioRad) for 1 hour. Tankyrase (Santa Cruz Biotech, Santa Cruz, CA), Axin2, active beta-catenin, beta-catenin, actin, CDC2 and CDK2 primary antibodies (Cell Signaling, Danvers, MA) were diluted at 1:1,000 in TBST containing 5% protease-free bovine serum albumin, and the membranes were incubated overnight at 4°C with rocking. After washing three times with TBST, the membranes were incubated for 1 h at room temperature with anti-rabbit or anti-mouse IgG horseradish peroxidase–conjugated antibody at a final dilution of 1:50,000 in TBST. After washing three times with TBST, bound antibodies were detected by enhanced chemiluminescence (Millipore, Billerica, MA).

### RT-PCR

RT-PCR was used to evaluate gene expression of the WNT dependent genes CD44, Axin2 and JAG1. Total RNA was extracted using the RNeasy Mini kit (Qiagen, Valencia, CA). cDNA was synthesized using the Applied Biosystems high capacity cDNA reverse transcription kit, following the manufacturer's instructions. Validated and pre-designed primer/probes for CD44, Axin2, JAG1 and the housekeeping gene GAPDH were purchased from Life Technologies (Carlsbad, CA). Samples were amplified using the ABI Step One Plus RT-PCR system. Relative expression of the mRNA analysed was estimated using the formula: 2^−ΔCT^, where Δ*C*_T_ = *C*_T_ (mRNA) – *C*_T_ (Housekeeper).

### Immunohistochemistry

Tumor tissues from control, AZ1366, irinotecan, AZ1366 + irinotecan treated groups were fixed in formalin immediately after surgical excision and processed into paraffin wax blocks. Sections were deparaffinized using standard histologic procedures, and an antigen retrieval method (pressure cooker and high EDTA buffer) was used to ensure optimal antigen integrity and expression. β-catenin staining was used to determine nuclear levels after treatment. The slides were scored (blinded) by a clinical pathologist.

### Proliferation and cell cycle analysis

The LoVo and RKO CRC cell lines were routinely cultured in RPMI 1640. All medium was supplemented with 10% FBS, 1% penicillin–streptomycin, and 1% MEM nonessential amino acids. All cells were kept at 37°C under an atmosphere containing 5% CO2. All CRC cell lines used in this study have been fully characterized and authenticated in the University of Colorado Cancer Center DNA Sequencing and Analysis Core. Cytotoxic effects on the LoVo and RKO CRC cell lines were determined using the sulforhodamine B assay (13). Briefly, cells in logarithmic growth phase were transferred to 96-well flat-bottomed plates with lids. Cell suspensions (100 μL) containing 500 to 3,000 viable cells were plated into each well and incubated overnight before exposure to varying concentrations of AZ1366 (5, 1, 0.5 μM) or SN-38 (16, 8, 4 nM) as single agents or in combination for 72 hours. After drug treatment, medium was removed and cells were fixed with cold 10% TCA for 30 minutes at 4°C. Cells were then washed with water and stained with 0.4% sulforhodamine B for 30 minutes at room temperature. The plates were washed with 1% acetic acid followed by stain solubilization with 10 mM Tris. The plate was then read on a plate reader (Biotek Synergy 2) set at an absorbance wavelength of 565 nm. For cell cycle analysis, cells (2 × 10^5^ per well) were seeded in six-well plates and allowed to attach for 24 hours. The cells were then treated with AZ1366 (5 μM), SN-38 (16 nM) or combination for 48 hours, washed in PBS, and resuspended in Krishanaposs stain. The cells were stained for 24 hours at 4°C before analysis by flow cytometry at the University of Colorado Cancer Center Flow Cytometry Core Facility.

### Statistical analysis

For single agent studies, PDTX explants were considered to be responsive to AZ1366 treatment if the tumors treated have at least a 80% decrease in growth relative to the control tumors, where tumor growth is measured by the tumor growth inhibition index (TGII), a standardized measure of tumor growth, which is calculated using the following formula: TGII = (tumor volume of TX on Day 28 – tumor volume of TX on Day 0)/(tumor volume of Con on Day 28 – tumor volume of Con on Day 0) * 100, where TX is the AZ1366 antibody-treated xenograft and CX is the control-treated xenograft. An unpaired Student *t*-test was used to determine whether the means between control and AZ1366 were significant at end of treatment (∼28 days). For combinational treatment, a one-way analysis of variance (ANOVA) was used to determine whether the means were significantly different overall at end of treatment. A logarithm transformation of the data was used to stabilize the variance. If the overall means were significantly different, we carried out a pair-wise comparison. *P* values were adjusted using Tukey's method for multiple comparisons. SE of the mean was indicated for each value by a bar. A student's *t*-test was used for comparisons between two groups (combo sens vs. no combo effects) for the evaluation of baseline tankyrase and NuMA protein levels as well as irinotecan TX/Con). All analyses were carried out using Graph-Pad Prism version 5.0c for Windows (GraphPad Software, San Diego) and SAS statistical software (SAS, Cary, NC).
